# Special Issue “Forensic Genetics and Genomics”

**DOI:** 10.3390/genes12020158

**Published:** 2021-01-25

**Authors:** Emiliano Giardina, Michele Ragazzo

**Affiliations:** 1 Department of Biomedicine and Prevention, Tor Vergata University of Rome, 00133 Rome, Italy; michele.ragazzo@uniroma2.it; 2 Genomic Medicine Laboratory UILDM, IRCCS Santa Lucia Foundation, 00179 Rome, Italy

The technological and scientific progress that we have experienced in recent years has contributed to characterization of the complex processes underlying human biology and evolution. In this regard, the studies performed on humans, both in pathological and physiological conditions, have been fundamental to improving knowledge of how genes, epigenetic modifications, aging, nutrition, drugs, and the microbiome affect the state of health and influence the onset of diseases [1]. Furthermore, it has been possible to identify several genes and/or variants responsible for variability in the response to pharmacological treatments in terms of safety and efficacy [1]. 

From a forensic field application point of view, the technological progress undergone by biology has allowed the development of innovative tools for scientific investigation. DNA analysis is no longer solely for comparative use but also for investigative use. In the last 30 years, alongside the development of increasingly sensitive techniques capable of typing biological samples consisting of even only a small number of cells, protocols and software have been developed for the interpretation of predictive markers of phenotypic, ancestral characteristics, and for the biostatistical evaluation of evidence [2,3].

The Special Issue “*Forensic Genetics and Genomics”* focuses on the latest scientific achievements in the field of forensic biology, from the introduction of new technologies allowing DNA analysis at crime scenes to the use of big data from genome sequencing studies and the study of population genetics for the development of protocols with investigative and phylogenetic purposes.

This Special Issue features eleven high-impact scientific articles. One of the most up-to-date issues is the possibility of analyzing DNA directly at the crime scene. A drawback of current forensic DNA technology is the need for cumbersome equipment, making it difficult to operate outside the laboratory environment. Transitioning forensic DNA analysis from the laboratory to the scene of an incident should deliver wide-ranging benefits in terms of the speed of result delivery, reduced contamination risk, and more efficient staff training [4,5]. Oxford Nanopore Technologies (ONT) developed the MinION, a long-read sequencer powered by USB. Its small size and portability make it ideal for remote analysis [4].

The article *Nanopore Sequencing of a Forensic STR Multiplex Reveals Loci Suitable for Single-Contributor STR Profiling* explores the possibility of using this new technology in detail, highlighting its current limitations though also proposing short-term solutions that could allow partial rapid application [4]. While the authors confirm that the technology is not ready, at this point, for immediate application in routine use, they have identified STR loci that ensure precise nanopore STR sequencing. Some characteristics such as repeat number, repeat pattern complexity, flanking region sequence, and the presence of homopolymers strongly influence successful genotyping. As a result, it was possible to design an STR panel that could be typed via nanopore sequencing, although further studies will be needed to validate this technology [4]. 

Continuing the topic of on-site genetic analysis, the work of Ragazzo M et al. *Comparative Analysis of ANDE 6C Rapid DNA Analysis System and Traditional Methods* compares the rapid analysis of DNA using the ANDE 6C with that of capillary electrophoresis [6]. The results met the ISO/IEC 17025 requirements, enabling ANDE 6C to receive accreditation for the rapid method. This means that rapid technology has certainly reached a level of reliability which has made its use in forensic genetic laboratories a reality [6].

In the field of new technologies, the article by Ragazzo et al. describes the application of NGS technology for DNA typing and for personal identification in the case of mixed biological evidence [5]. The results confirm that NGS technology offers a huge range of additional information on samples but cannot ensure a higher sensitivity with respect to traditional methods [7]. Thus, the Precision ID GlobalFiler™ NGS STR Panel v2 is confirmed as a powerful method for kinship analyses and typing reference samples, but its use in analyzing biological evidence should be carefully considered on the basis of the characteristics of the evidence (DNA amount and degradation) [7].

A number of papers on population genetics have been published in this Special Issue [8,9,10,11,12]. These findings are crucial as they determine the theoretical foundations of the studies on ancestral information and migration. These articles have, in fact, investigated some aspects of population genetics using different markers, such as ancestry informative single nucleotide polymorphisms (AISNPs) and markers located on the Y chromosome and mtDNA [8,9,10,11,12].

The paper *Ancestry Prediction Comparisons of Different AISNPs for Five Continental Populations and Population Structure Dissection of the Xinjiang Hui Group via a Self-Developed Panel* selected 30 novel AISNPs able to discriminate between African, European, East Asian, and South Asian populations and developed a multiplex analysis based on the NGS platform, comparing the resulting ancestry resolutions with those provided by the other published AISNPs [8,13,14,15]. After a cross-validation procedure, they evaluated the panel in the Xinjiang Hui group [8]. The resulting population data in the Xinjiang Hui group can be used as a reference sample for inferring ancestry of the Hui group. Furthermore, population genetics analysis based on these 30 AISNPs of the Hui group compared with other continental populations revealed that the Hui group may have similar ancestry origins with East Asian populations [8].

The paper *Joint Genetic Analyses of Mitochondrial and Y-Chromosome Molecular Markers for a Population from Northwest China* reports an interesting study of the Chinese population [9]. China consists of 56 official ethnic groups, some of which include the Uygur, Kazak, Uzbek, and Kyrgyz groups living in northwest China [9]. The genetic diversities were analyzed based on 60 mtDNA loci and 24 Y-STRs for the Kyrgyz group in China [9]. The haplogroup distributions for these markers demonstrated the differing genetic backgrounds of this group in terms of maternal and paternal lineages [9]. Thus, mtDNA analysis revealed that the Kyrgyz group is closer to East Asian populations than they are to those in Europe [9]. However, Y chromosome markers provided different results, suggesting differences between the maternal and paternal inheritances. Further studies will be needed to test a higher number of markers and to address the effects of post-marital residence [9]. 

The paper *Characterizing Y-STRs in the Evaluation of Population Differentiation Using the Mean of Allele Frequency Difference between Populations* describes the use of the mean of allele frequency differences (mAFD) from the Yfiler set and Yfiler Plus to determine any population sub-division [10]. These results showed that mAFD is suitable for characterizing Y-STRs in the evaluation of population differentiation [10].

The article *Genetic Reconstruction and Forensic Analysis of Chinese Shandong and Yunnan Han Populations by Co-Analyzing Y Chromosomal STRs and SNPs* reports a comparative study through the use of Y-STRs and low-resolution Y-SNPs in two Chinese populations—Shandong Han and Yunnan Han—to characterize the patrilineal patterns within these populations [11]. As a result, the phylogenetic reconstruction at an individual level, using a Y chromosome database, confirmed that Y-STRs combined with Y-SNPs can increase the power of discrimination of male pedigrees [11].

The paper *A Highly Polymorphic Panel Consisting of Microhaplotypes and Compound Markers with the NGS and Its Forensic Efficiency Evaluations in Chinese Two Groups* evaluates the use of a selection of 29 compound markers that are a combination of one InDel and one SNP in a genomic region [10]. In particular, they evaluated the ability of these genetic markers to detect the DNA mixture [12]. This preliminary report, while confirming that most of these 29 loci were relatively highly polymorphic in different continental populations, demonstrated that the power of these loci in detection of biological mixtures needs to be evaluated further [12].

The paper *The STRidER Report on Two Years of Quality Control of Autosomal STR Population Datasets* reports the two-year experience of STRidER, the STRs for the Identity ENFSI Reference Database [16]. It is a free, publicly available, high quality, online allele frequency database of STRs developed under the endorsement of the International Society for Forensic Genetics [14]. The report highlights the importance of the quality filter of STRidER prior to the publication of autosomal STR allele frequency data [16]. It is well known that there is a large proportion of errors in the datasets available for forensic as well as diagnostic purposes [16]. Data accuracy is fundamental in forensic genetics and the availability of rigorous data quality control can improve the precision of these databases [16].

Criminal DNA databases are expanding around the world to support the activities of criminal justice systems. The paper *Autosomal STR Profiling and Databanking in Malaysia: Current Status and Future Prospects* details the progress of DNA profiling and DNA databanking in Malaysia [15]. The article describes the main rules governing the use of DNA in forensics in Malaysia and discusses the main issues that need to be addressed to make the DNA database for forensic purposes even more effective [17]. 

The paper *Challenges in Human Skin Microbial Profiling for Forensic Science: A Review* reports on the possible applications of microbiome analysis in the field of forensic science. It is well known that humans have an extremely diverse microbiome that can be useful in inferring ethnicity and personal identification [18]. The forensic efficacy of microbial profiling for the identification and/or the association of individuals with criminal activities will depend on technological evolutions both to limit the variability of results and to achieve a greater level of standardization. A lot of further work is required but the preliminary findings are fascinating [18].

In conclusion, the heterogeneity and quality of the works presented in this Special Issue represent the constant advancement of knowledge in the field of forensic genetics.

The development of technologies that allow the analysis of huge portions of the genome or the whole genome is already promoting the transition between forensic genetics and forensic genomics. Alongside the classic typing of STR markers for personal identification and for the genetic characterization of biological evidence, we are witnessing the development of methods that analyze every element of the genome, the transcriptome, and the epigenome. The new achievements of omic sciences can be used in forensic sciences as long as technological evolution allows the validation and standardization of the results. We are likely at the dawn of a new forensic genomics era whose potential applications are limited only by the imagination of researchers.
